# Effects of Climate Change on Vegetation Cover in the Oued Lahdar Watershed. Northeastern Morocco

**DOI:** 10.3390/plants10081624

**Published:** 2021-08-06

**Authors:** Hind Khalis, Abdelhamid Sadiki, Fatimazahra Jawhari, Haytam Mesrar, Ehab Azab, Adil A. Gobouri, Muhammad Adnan, Mohammed Bourhia

**Affiliations:** 1 Laboratory of Geosciences, Environment and Associated Ressources LGERA, Faculty of Sciences, University Sidi Mohamed Ben Abdellah (USMBA), Fez 30000, Morocco; abdelhamid.sadiki@usmba.ac.ma (A.S.); mesrar.haytam@gmail.com (H.M.); 2 Laboratory of Biotechnology and Preservation of Natural Resources (BPRN), Faculty of Sciences, University Sidi Mohamed Ben Abdellah (USMBA), Fez 30000, Morocco; fatimazahra.jawhari@usmba.ac.ma; 3 Department of Nutrition and Food Science, College of Science, Taif University, P.O. Box 11099, Taif 21944, Saudi Arabia; e.azab@tu.edu.sa; 4 Department of Chemistry, College of Science, Taif University, P.O. Box 11099, Taif 21944, Saudi Arabia; a.gobouri@tu.edu.sa; 5 Department of Botanical and Environmental Sciences, Faculty of Biological Sciences, Kohat University of Science and Technology, Kohat 26000, Khyber Pakhtunkhwa, Pakistan; ghurzang@hotmail.com; 6 Laboratory of Chemistry-Biochemistry, Environment, Nutrition and Health, Faculty of Medicine and Pharmacy, Hassan II University, Casablanca 5696, Morocco; bourhiamohammed@gmail.com

**Keywords:** climate change, droughts, desertification, land cover degradation, NDVI, LST

## Abstract

Episodes of drought that Morocco experienced in the years 1984–1986, 1993–1995, and 1997–2000 had repercussions that were felt many years later and continue to pose serious problems for environmentalists, as some of the affected lands have become practically deserted. These problems acted on the socio-economic conditions and created severe constraints for the development of the country. This work was conducted to study and identify changes that occurred in vegetation cover in the Oued Lahdar watershed (Rif, Morocco) between 1984 and 2017 using Land Surface Temperature (LST), Normalized Difference Vegetation Index (NDVI), Landsat TM 5, and Landsat OLI 8. The LST had significantly increased overall from 1984 to 2017, where it moved from a mean value of 29.4 °C in 1984 to 40.4 °C in 2007 and then reduced slightly to 37.9 °C in 2017. The vegetation cover index for the study area indicates that in 1984, fully vegetated areas represented 94.3% before deteriorating to 35.4% in 2007 and recovering in 2017 to 54.3%. While bare soil, which previously constituted 5.7%, reached a very high value of 64.6% in 2007 and then decreased to 47.7%. This study contributes towards society as it provides interesting data about the consequences of climate change in the area studied as well as potential protective strategies to protect vegetation cover.

## 1. Introduction

Morocco’s climate varies considerably from north to south. Rainfall and temperature are strongly influenced by the Atlantic Ocean in the west, the Mediterranean Sea in the north, the Sahara Desert in the south and southeast, and in particular by the position of the Atlas and the Rif mountains. This geographical and topographical characteristic of Morocco determines the distribution of rainfall over the country.

The increasing temperature in Morocco is associated with global climate change. The year 2017 was the hottest year on record, surpassing the previous high mark set in 2016. Similar trends have been recorded for Morocco and the Middle East and North Africa region, though there are year-to-year country-level variations from the global mean [1]. With approximately global temperatures of 1.2 °C above pre-industrial levels.

Due to severe climatic conditions, deforestation, overgrazing, and poor planting techniques, more than 40% of the total area of Morocco have been affected by soil erosion. Climate has a direct effect on soil erosion through rainfall, runoff, and indirectly through vegetation. As a result, there is a strong link between vegetation, soil, and the phenomenon of degradation. Plant cover is an important factor in protecting soil from erosion by maintaining soil fertility and moisture. It can also play an important role in thermal protection: maximum temperatures under canopy cover are generally 2 to 4 °C lower than on surrounding agricultural land, and minimum are 1 to 2 °C higher [2]. The role of thermoregulation played by vegetation covering river embankments was reported as follows: soil temperatures were almost 4 °C higher for vegetated embankments than for bare embankments. The destruction of vegetation leads in the long term to soil degradation and aggravated cases to desertification.

Rouse and his collaborators [3] developed the NDVI (Normalized Difference Vegetation Index) using the red and near-infrared bands of the electromagnetic spectrum. The Vegetation Index reveals that the vegetation covers relative to the ground. It is mainly correlated with leaf chlorophyll activity. Indeed, chlorophyll pigments absorb strongly in the red and leaves reflect in the near-infrared. NDVI was used for the diagnosis and evaluation of the active vegetation. The spectral signature of plants ranges from a very active photosynthetic green state in wet weather to a very weak active dry state in dry weather, which has no distinct spectral signature to differentiate it from bare soil. High values indicate abundant and dense vegetation. These values progressively decrease as chlorophyll activity and vegetation decline. Several studies [4,5,6] have been devoted to linking this index to a land cover structure parameter (percent land cover, green biomass, or leaf area index). LST is the main topic for developing methodologies to be measured from space. LST is an important factor in many study areas, such as global climate change, hydrological and agricultural processes, and urban land use/land cover [7].

The higher NDVI index indicates the presence of vegetation, i.e., the quantity or state of vegetation. Land surface temperature (LST) is one of the key factors in the physical process of the Earth’s surface, combining surface-atmosphere interactions and energy flows between the atmosphere and the ground [8,9]. The lowest LSTs are generally found in areas with high NDVI. This negative correlation between NDVI and LSTs is valuable for urban climate studies found that the vegetation fraction is slightly more negatively correlated with LSTs.

Many researchers have tried to understand the increase in temperature in the world. Few studies have involved the continuous rise of temperature [10,11], while few others analyzed the relationship between LST-NDVI over Morocco, Europe, and North America [4,6,12].

In Morocco, the arid zones represent 77% of the national territory and the Saharan regions alone occupy 60%. However, if we consider the arid zone in the broad sense, including the semi-arid regions where the risks of desertification are high, we end up with 90% of the territory [13]. The region of Taza had become arid between 1961 and 2008 (according to the aridity index of de martonne) [14].

The present work was conducted to investigate the evolution of the vegetation state in the Oued Lahdar watershed between 1984 and 2017, and its effect on land surface temperature increase (LST). During these years the soil recorded the effects of major drought episodes experienced in various regions of Morocco since the 1980s.

A literature review on drought in Morocco indicates that occurrences of drought during the 20th century grew steadily [13,15,16]. The periods of drought were marked by the rise in soil temperature. The study by remote sensing of this index during 33 years allows us to contribute to diagnosing the degradation of the vegetation during the last decades. This method was used for the first time in Morocco and could contribute to combatting desertification, of which Morocco has a particularly rich history, which began in 1951.

### 1.1. Study Area

The Riffian domain is bordered by the Mediterranean Sea on more than 400 km of coastline and in its northwestern part, by the Atlantic Ocean, constitutes the most septentrional part of Morocco. Geologically, it is the only Moroccan massif resulting from the alpine orogeny, most of its facies having more affinities with those of Andalusia than with those of the rest of Morocco. Geographically, the reliefs are strong and uneven, with varied geological nature of the terrain (schists, marl, sandstone, limestone, etc.) [14,15]. This leads in particular to great variability in the potentiality of groundwater resources, and a dissymmetry of the hydrography between the Mediterranean slope (short wadis with very steep slopes) and the Atlantic slope (long wadis, with many tributaries).

**Figure 1:**
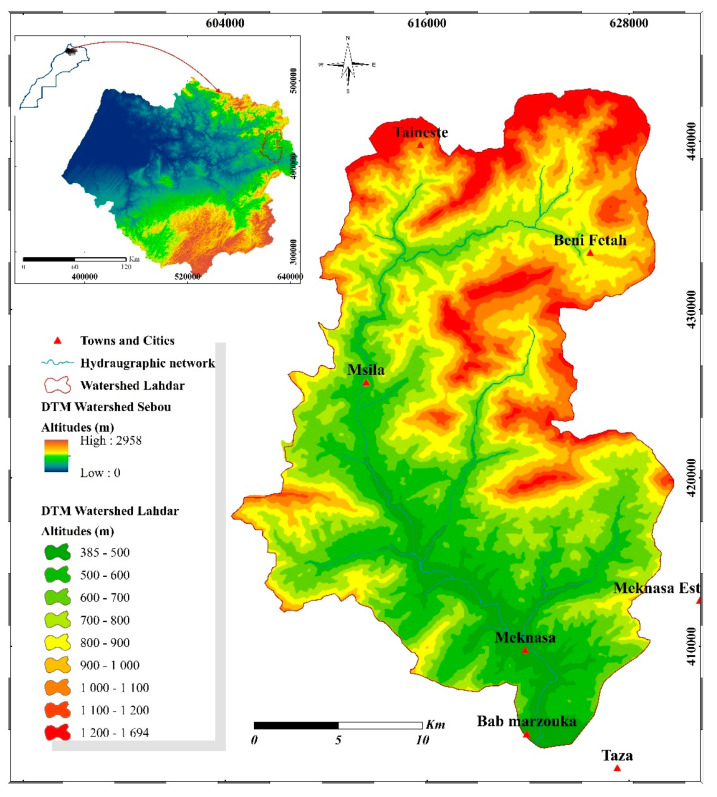
The geographic situation of the Study Area.

The Oued Lahdar watershed is part of the Oued Inaouene watershed, which is itself a sub-basin of the Sebou (Figure 1), covering an area of 3680 km^2^. The Oued Inaouene is considered the second main tributary of the Sebou after the Oued Ouergha; it flows in an east-west direction along the southern Rif corridor, towards the Idriss I dam. It thus covers part of the External Rif in its left part, and part of the Middle Atlas in its right part, and its valley the southern Rif corridor Fez-Taza. This geographical position of the watershed of Oued Inaouene presents a very marked dissymmetry due to the contrast of the lithology of its two slopes: the southern limestone and dolomitic slope (Middle Atlas) presents harder facies than the northern slope (Rif) consisting of marly hills.

Oued Lahdar originates in the mountains of the Outer Rif, in the northern part of Oued Inaouene. The watershed of Oued Lahdar extends over the Outer Rif, which is characterized by a fragile terrain and high altitudes. The climate that dominates in the watershed is the Mediterranean with a continental oceanic influence. The average annual rainfall of the watershed varies between 600 mm and 800 mm/year.

In terms of vegetation, the Rifain region, due to its differences in altitude, facies, and humidity, offers a great diversity in the natural population and the crop range. The shrubby vegetation is represented in the western half of the Rif by the dwarf palm, or doum (*Chamaerops humilis*) and the mastic tree (*Pistacia lentiscus*), which combined with other species form an impenetrable scrubland. The eastern half is characterized by the jujube tree (*Zizyphus lotus*) esparto (*Stipa ten acissima*). These areas correspond roughly to those of the olive tree (replaced in altitude by the walnut tree) in the West and the almond tree in the East. The forest formations, which are generally very discontinuous, are mainly characterized by the following species: cork oak (*Quercus suber*) on the acid soils of the peninsula, holm oak (*Q. ilex*), and *Q. pyrenaïca* in the central mountainous areas with maritime pine (*Pinus pinaster)*. Finally, the degraded soils that receive low rainfall are the domain of thuja (*Callitris articulara*), and Aleppo pine (*Pinus halepensis*).

As far as crops are concerned, the mountains have small, and often irrigated fields with supplementary livestock farming (usually goats), while the hills, particularly in the Prerif, have cereal crops. The irrigable plains bear market gardening crops.

### 1.2. Satellite Data

This study was carried out using satellite images coupled with detailed field observations, verification, and statistical analysis to assess the relationship between these variables. The satellite images were collected from the USGS data portal [16]. A Landsat TM 5 (Thematic Mapper) satellite image dated 1984, 1994, 2005, and 2007, and Landsat OLI 8 (Operational Land Imager) dated 2016 and 2017. Satellite image pre-processing: Following the reading of the raw images, a generalized and systematic pre-processing phase was required. The pre-processing applied to the images included the following procedures: radiometric calibration and radiometric correction Fast Line-of-sight Atmospheric Analysis of Hypercubes (FLAASH). As we chose to map only natural vegetation and to exclude cultivated plants, the period chosen for the NDVI calculation was June/July. This choice was justified for LST by the fact that this period corresponds to the maximum temperature in Morocco.

Landsat series data are downloadable free of charge directly from the United States Geological Survey (USGS) website. The characteristics of the Landsat TM 5 and OLI 8 satellite sensors used in this study are presented in Table 1.

### 1.3. Meteorological Data

**Figure 2:**
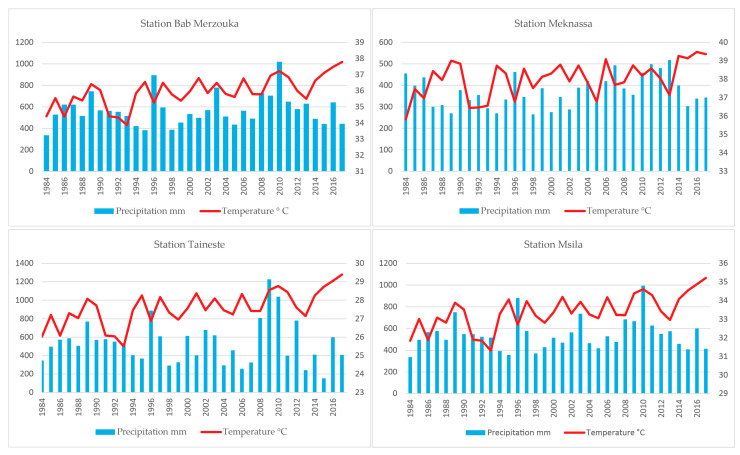
Mean annual precipitation (primary *y*-axis, blue histogram) and temperature (secondary *y*-axis, red curve) on the main stations of the Oued Lahdar Watershed for the period 1984–2017.

Monthly and annual average precipitation and temperature data are calculated over the period 1984 to 2017. Rainfall patterns throughout the basin are very similar: heavy rainfall in winter, relatively minimum in autumn and spring, and very light rainfall in summer. The average annual rainfall increases from 383 mm at Meknassa, to 575 mm at Taza, 577 mm at Bab Merzouka. The increase in rainfall downstream can be explained by the influence of the Middle Atlas (Figure 2).

## 2. Methodology

### 2.1. Image Corrections

The images were radiometrically normalized to reduce any variations in the signal received by the sensor. Afterward, atmospheric corrections FLAASH for multi-temporal optical satellite images are necessary to control change detection analyses such as normalized difference vegetation index (NDVI) rationing [17].

### 2.2. Normalized Difference Vegetation Index (NDVI)

The NDVI represents two environmental factors: the ecosystem, which explains long-term changes in vegetation as well as the ecosystem in response to climate fluctuations. The purpose of using the vegetation index has several objectives between them we chose; Estimation of the mass of green plants covering the soil [18]. The evolution of land cover on a temporal scale [19].

To determine the NDVI in the Oued Lahdar watershed, we used the Landsat 5 TM and Landsat-8 OLI satellite series (This study was carried out using satellite images coupled with detailed field observations, verification, and statistical analysis to assess the relationship between these variables. The satellite images were collected from the USGS data portal [16]. A Landsat TM 5 (Thematic Mapper) satellite image dated 1984, 1994, 2005, and 2007, and Landsat OLI 8 (Operational Land Imager) dated 2016 and 2017. Satellite image pre-processing: Following the reading of the raw images, a generalized and systematic pre-processing phase was required. The pre-processing applied to the images included the following procedures: radiometric calibration and radiometric correction Fast Line-of-sight Atmospheric Analysis of Hypercubes (FLAASH). As we chose to map only natural vegetation and to exclude cultivated plants, the period chosen for the NDVI calculation was June/July. This choice was justified for LST by the fact that this period corresponds to the maximum temperature in Morocco.

Landsat series data are downloadable free of charge directly from the United States Geological Survey (USGS) website. The characteristics of the Landsat TM 5 and OLI 8 satellite sensors used in this study are presented in Table 1.

(1) $$\mathrm{NDVI}=\frac{\left(\mathrm{NIR}-\mathrm{RED}\right)}{\left(\mathrm{NIR}+\mathrm{RED}\right)}$$

NIR = near-infrared

The LST is the radiative temperature of the ground. It influences the partition of energy between ground and vegetation and determines the surface air temperature [20,21].

The effective at-sensor brightness temperature (BT) also known as black body temperature is obtained from the spectral radiance using Plank’s inverse function [22].

Conversion of digital number (DN) to Spectral Radiance L_λ_ [23].

(2) $$L_{\lambda }=L_{\mathrm{MIN}}+\left(L_{\mathrm{MAX}}-L_{\mathrm{Min}}\right)\times \frac{\mathrm{DN}}{255}$$

L_λ_ = Spectral radiance,

L_MIN_ =Spectral radiance of DN value 1

L_MAX_ = Spectral radiance of DN value 255 DN = Digital Number

Conversion of spectral Radiance to temperature in Kelvin [22,24].

(3) $$B_{T}=\frac{K_{2}}{\ln\left(\left(\frac{K_{1}}{L}\right)+1\right)}$$

K_1_ = Calibration constant 1

K_2_ = Calibration constant 2

B_T_ = surface temperature

The calibration constants K_1_ and K_2_ obtained from the Landsat data user’s manual are given in Table 2.

In Table 3, LST classification is based on the mean temperature (Tmean) and the standard deviation (STD) of the six years of the study.

### 2.3. Statistical Analysis

The statistical analysis was performed by using LST and NDVI data, which includes central tendency, variability, and shape measurements. Next, the analysis of variance ANOVA for the variables (LST, NDVI) was conducted to study the evolution of variable factors over time RED.

## 3. Results & Discussion

### 3.1. Plants Inventory

The determination of plant diversity was carried out by analyzing the exhaustive inventories of species in the study area. Botanical identification was carried out according to the earlier literature [26,27,28]. The reference specimens of each plant were deposited in the herbarium of the Laboratory of Biotechnology, Environment, Agri-food, and Health, University of Sidi Mohamed Ben Abdellah, FSDM-Fez, Morocco.

The watershed of the Oued Lahdar presents a bioclimatic stratification composed of about 36 species belonging to different families (Table 4). The most represented families are Lamiaceae, Pinaceae, Asteraceae, and Cupressaceae.

According to the model used to study the ecology of plants in [29]. The Oued Lahdar watershed has a mountainous exposure with similar characteristics to the study area of Farhad Aghajanlou, such as sandstone and limestone. This study helped us to characterize the different plant species that exist in the watershed of Oued Lahdar.

**Figure 3:**
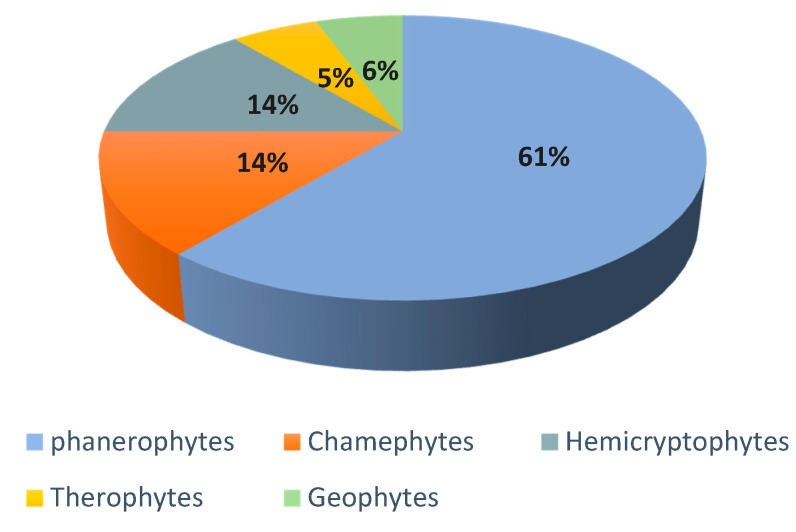
Biological types of plant species encountered in the study area.

Analysis of the biological type of species inventoried in the area (Figure 3) shows that the proportion of the different categories of biological types is variable, with the dominance of phanerophytes (61%), followed by chamaephyte and hemicryptophytes (14%), while the lowest percentage is those of geophytes (6%) and therophytes (5%).

**Figure 4:**
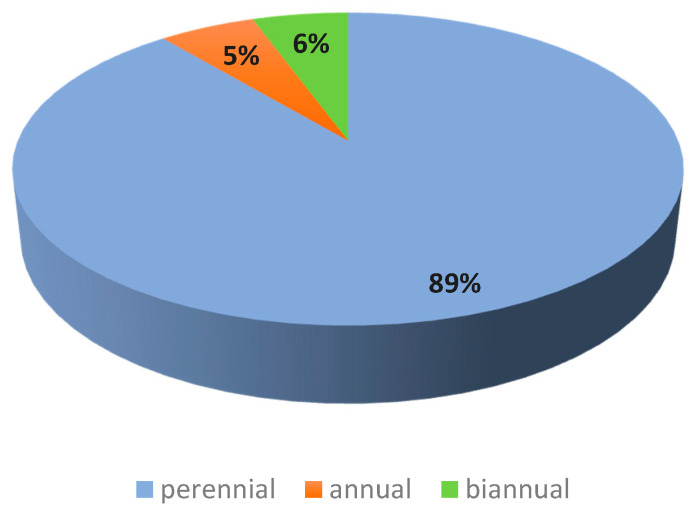
Lifespan of plant species encountered in the study area.

In the study area, perennials dominate with a percentage of 89%, while annuals and biennials represent only 5% and 6%, respectively (Figure 4).

### 3.2. Cartographic Analysis

#### 3.2.1. Normalized Difference Vegetation Index

**Figure 5:**
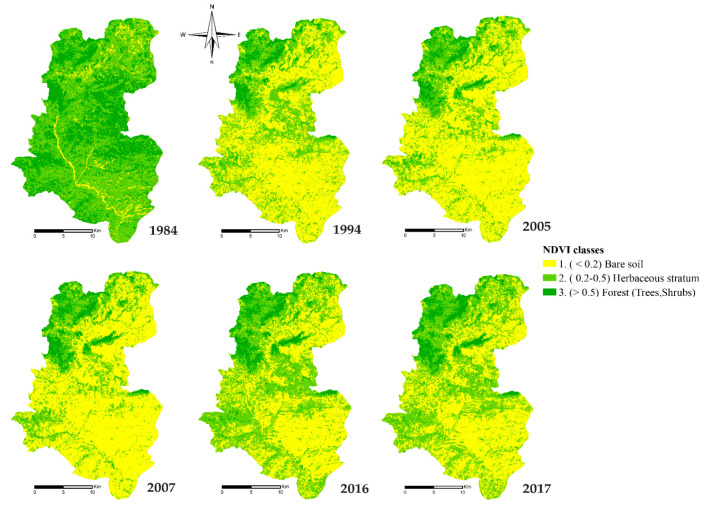
Monitoring of cover vegetation in the Oued Lahder watershed from 1984–2017.

The NDVI map analysis (Figure 5) reveals that in 1984, the lowest value of NDVI found was only 5.7% of the catchment area in streams and bare soils in small areas. 63.2% of the study area presented an average NDVI corresponding to sparse vegetation such as grassland or senescing crop and shrubs. High NDVI testifying of forests and dense vegetation covered 31.1%.

In 1994 the natural vegetation cover was too affected. Bare soil reached 57.7% of the study area. The sparse vegetation layer was gradually reduced to 36.9%. 5.4% of the remaining area was a forest. More than perennial cover was also eliminated during the year due to low rainfall.

In 2005, bare soil reached 62.6% of the total area and became 64.6% in 2007. The sparse vegetation layer was reduced to 31.1% and then 28.2% in 2007. The forests gradually increased to 6% in 2005 and 7.2% in 2007. The decline in plant cover continues to increase with a slight increase in the area of forests.

In 2016, we could note that the bare soil surface was reduced to 45.3% and the sparse vegetation layer significantly increased to 46.3%. The presence of dense vegetation was also improved in the Northwest zone by 8.3%.

The last year of our study was 2017, in which it was reported that the bare soil extended by 47.7%, the sparse vegetation layer reduced by 44.4%, and the forests 7.9% of the watershed of Oued Lahdar.

#### 3.2.2. Land Surface Temperature

**Figure 6:**
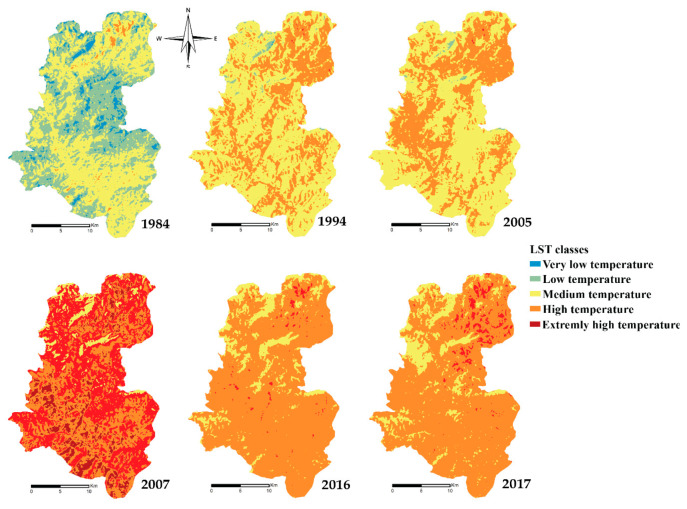
Monitoring of LST changes over the years from 1984–2017.

Geographic studies for the LST (Figure 6) show that in 1984, the temperature in the study area varied as follows: very low temperature filled 4.2%, low temperature covered 43.3%, medium temperature occupied 51.3%, and high temperature covered 1.2% (Figure 6).

In 1994 the very low-temperature area disappeared from the watershed, while the areas with the low temperature decreased to be 1.2% only. On the other side, the area medium-temperature increased to 63.9% and those with high temperatures to 34.9%of the watershed area.

In 2005, the temperature in the study area varied as follows: the low-temperature area showed 0.5%, where the area with medium temperature remained almost the same presenting 59.2%, the areas with high temperatures covered 40%, and the very high temperature displayed only 0.1% of the study area.

The year 2007 presented exceptional results, where the medium temperature zone presented 5.6%, the high-temperature zone 48%, and the very high-temperature zone 37.7%. A new category appeared that year, namely the extremely high-temperature category, with 8.7%.

The temperature decreased significantly in 2016 and 2017; the medium temperature zone covered 12.6% in 2016 and 14.2% in 2017. The high-temperature zone covered between 82.2% in 2016 and 86.1% in 2017, while the very high-temperature zone was between 1.2% and 3.6%.

### 3.3. Statistical Analysis

#### 3.3.1. Summary Statistics of LST and NDVI

ANOVA and simple regression were used for statistical analysis for a better understanding of the evolution of NDVI and LST over time.

Table 5 shows the summarized statistics for each selected data variable (LST, NDVI) by representing the measurements recorded in June of the six years considered, the measurements of average, variability and shape, and the shape measurements. Standardized Skewness γ1 and standardized Kurtosis γ2 were used to determine whether the sample has a normal distribution. The standard normal distribution is perfectly symmetrical when Skewness and Kurtosis are equal to 0. Values outside the range of −2 to +2 indicate a significant deviation from normality.

In this case, the measurements of Standardized Skewness for LST γ_1_ = −0.24 means an asymmetry on the left. For NDVI γ_1_ = 0.53 means positive Skewness and indicates an asymmetry on the right. The measurements of standardized Kurtosis show values in LST γ_2_ = 0.21 and in NDVI 0.77 that may be classified as Mesokurtic distributions.

#### 3.3.2. Simple Regression-LST in the Function of NDVI

The adjusted model R^2^ explains 78.21% of the variability in LST. The correlation coefficient is −0.88, indicating a moderately strong negative relationship between the variables (Table 6). The standard error of estimation indicates that the standard deviation of the residues is 3.62. The average absolute error of 2.63 is the average value of the residues. The Durbin-Watson (DW) statistic allows determining if there is a significant correlation based on the order in which they appear in the data file. Since the probability value is less than 0.05, this indicates a possible serial autocorrelation of the residues at the 95.0% confidence level. Display the residues with the observation numbers to see if there is a particular shape in the graph.

Since the probability value in the ANOVA Table 6 is less than 0.05, there is a statistically significant relationship between LST and NDVI at the 95.0% confidence level.

**Figure 7:**
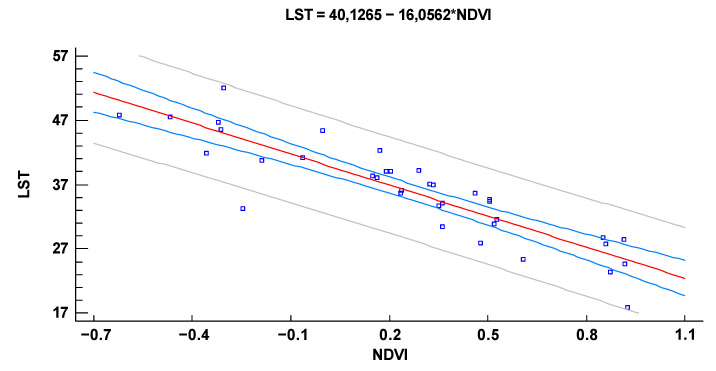
A-Scatterplot of LST and NDVI, B- Plot of the simple linear regression between NDVI and LST.

The output shows the results of fitting a model to describe the relationship between LST and NDVI (Figure 7). The equation of the fitted model is

(4)  LST=40.1265−16.0562×NDVI

These results confirm those obtained by using ANOVA and help explain the causes of environmental degradation.

## 4. Vegetation and Temperature Interannual Variability

Statistical analysis indicates a strong relationship between Land Surface Temperature (LST) and vegetation index (NDVI). This can help us conclude that, over time, the temperature increases when and where the vegetation cover decreases, and vice versa.

The study area is characterized by the presence of cultivated and wild plant species. However, since 1984, the vegetation cover of the study area has decreased significantly due to habitat fragmentation, overgrazing, deforestation, desertification, and other anthropic and climatic threats. This has a significant effect on the reduction of the area covered, and on the increase in temperature. The NDVI study reveals this alarming degradation of the vegetation

**Figure 8:**
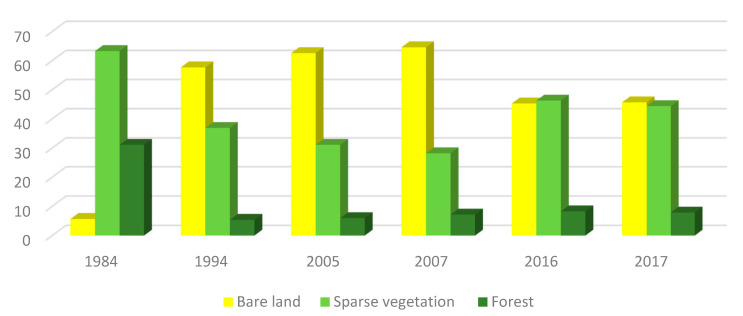
Evolution of different land cover between 1984 and 2017.

Between 1984–1994 (Figure 8), natural vegetation covered 94.3% of the study area before being decreased to 43.3% in 1994, and the forest covering 31% of the watershed was reduced to 5%. The sparse vegetation such as grasslands or senescent shrubs and shrubs covered 63.2% was reduced to 36.9%. The bare ground, which affected 5.7% at earlier times, reached 47.7% in 2017.

Between 1994 and 2007, the vegetation cover continued to deteriorate progressively. Bare soil area increased to 64.6%, sparse vegetation continued to decline by 28.2%, even though the forest improved slightly by 7.2%.

From 2006 to 2009 within the framework of the LIFE-Pays-Tiers program (mainly Mediterranean and Baltic Sea countries), funded by the European Union. At the Moroccan level, the CRTS (Royal Center for Spatial Remote Sensing), the MAPM (Ministry of Agriculture), and the HCEFLCD (High Commission for Water, Forests and the Fight against Desertification) [30], were involved in the nature conservation project, regeneration and forest management. It is thus fitting that the vegetation cover had improved noticeably between 2007 and 2017 in the study area.

In 2016, we can note that the bare soil area was reduced by 45.3% and the sparse vegetation layer significantly increased by 46.3%. Dense vegetation and forest also improved in the northwest area by 8.3%. Between 2016 and 2017, there was a slight deterioration, where the bare soil increased by 2.7%, the sparse vegetation layer decreased by 1.9%, while forests increased by 0.4% of the Oued Lahdar watershed.

The slight improvement observed can be explained by the exceptionally rainy years from 2008 to 2010 that had never been known since dry years similar to that of the 80s (INRA). In addition, Morocco adopted a National Action Program to Combat Desertification (NAP-CD) in June 2001 [31]. As a result, several integrated projects have been implemented for the development of forest areas and arboriculture, in particular olive trees, the development of watersheds, rain-fed agricultural land, and rangelands, which in our study area can be explained by the increase in forests by 8% in 2016. Besides, stricter control of water, forest management, and agriculture on natural resources (water, forests, soils, etc.).

**Figure 9:**
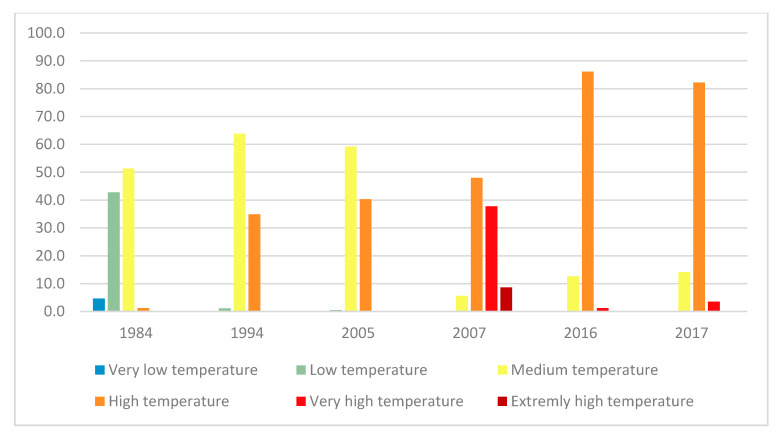
Evolution of different land surface temperatures between 1984 and 2017.

The geographical studies of the LST (Figure 9) show that between 1984 and 1994, the temperature in the study area (the Oued Lahdar watershed) varied as follows: the low temperature covered 42% and decreased to 1.2% in 1994, the medium temperature covered 51% and became 63.9%, the high temperature covered only 1.2% before being increased to 34.6% later.

Between 1994 and 2007, the normal temperature area decreased continuously to 0.5% in 2005, while the medium and high-temperature areas were 59.2% and 40.3% of the total study area respectively.

The year 2007 witnessed an alarming increase in temperature in the entire study area, with the medium temperature covered 5.2%, the area with high-temperature presented 48%, where the one with very high covered 37.7%, and the extremely high temperature appeared with 8.7%. In 2007, the increase in LST reached maximum values, resulting from the combination of the last droughts recorded in the 1980s, 1990s, and the early 2000s and global warming of the planet.

From the years 2008–2010 which were exceptionally rainy, the LST started to drop, and also there was no significant drought after 2010. Areas with very high temperatures decreased (from 37.7% in 2007 to 1.2% in 2016 and 3.6% in 2017) in favor of areas with high temperatures, which increased from 48% to 86.1% and 82.2% in the same order. Areas with medium temperature increased from 5.6% to 12.6 % and 14.2% in the same order. The decreasing of LST still didn’t reach the normal state in the watershed Oued Lahdar.

## 5. Conclusions

This study explored the spatial and temporal relationship between LST and NDVI over the Oriental Rif of Morocco. LST and NDVI investigated over the last 33 years showed that the study area can be considered as a fragile state of the Mediterranean environment, which was impacted by multiple anthropogenic and climatic degradation factors.

From 1984 to 2017, the land surface temperature had increased from 1984 to 2007 due to droughts, while a gradual decrease was registered after 2007 due to the partial restoration of vegetation in the Watershed after the rainy years 2008–2010. This finding could reflect the effect of vegetation cover thermoregulation.

In Morocco, the rainy years after 2008 have reduced the negative effects of drought, and therefore, they have played a crucial role in the restoration of vegetation, which is showing good results in the Watershed of the Oued Lahdar.

In comparison with diachronic analysis, on aerial photographs between 1986 and 2006, authors assessed forest decline at the equivalent of 5000 ha/year [15]. Another study reported the vegetation cover on Oued Sra watershed, neighboring Lahdar, showed that the forest cover which occupied 38% in 1986, decreased to 19% in 2006 and 15% in 2013 [32]. Furthermore, in the Taounate region, forest cover decreased by 11.76% from 1982 to 1992 [33].

The monitoring of land cover time series provides an overview of the increase or decrease of vegetation, while if we combine them with LST time series, they can become a good characterization of droughts in terms of identification and monitoring sensitive areas to be protected before reaching the desertification.

## Figures and Tables

**Table 1:** TM5 and Landsat 8 thermal bands.

Date of Images	Landsat Imageries	RED	NIR	Path/Row
8 June 1984	Landsat TM5	Band 3	Band 4	200/36
20 June 1994				
18 June 2005				
10 July 2007				
20 June 2016	Landsat OLI 8	Band 4	Band 5	
5 July 2017				

**Table 2:** TM5 and Landsat 8 thermal band calibration constants.

Image	Bands	K_1_ (w/(m^2^ sr lm)	K_2_ (K)
Landsat 8 OLI	Band 10	774.88	1321.08
	Band 11	480.89	1201.14
Landsat 5 TM	Band 6	607.76	1260.56

**Table 3:** Classification of the surface temperature [25].

LST Class	Class Range
Very low temperature	T ≤ Tmean − 1.5 STD
Low temperature	Tmean − 1.5STD < T ≤ Tmean − STD
Medium temperature	Tmean − STD < T ≤ Tmean
High temperature	Tmean < T ≤ Tmean + STD
Very high temperature	Tmean + STD < T ≤ Tmean + 1.5 STD
Extremely high temperature	T > Tmean + 1.5 STD

**Table 4:** An exhaustive inventory of plant species encountered in the study area.

Family	Scientific Name	Biological Type	Morphological Type
Anacardiaceae	*Pistacia atlantica* Desf.	Phanerophytes	Perennial
	*Pistacia lentiscus* L.	Phanerophytes	Perennial
Apiaceae	*visnaga daucoides* Gaertn.	Therophytes	Annual
Apocynaceae	*Nerium Oleander* L.	Phanerophytes	Perennial
Arecaceae	*Chamaerops humilis* L.	Phanerophytes	Perennial
Asparagaceae	*Urginea maritima* L. *Baker*	Geophytes	Perennial
Asteraceae	*Silybum marianum* Gaertn.	Hemicryptophytes	Biannual
	*Scolymus hispanicus* L.	Hemicryptophytes	Biannual
	*Calendula arvensis*	Therophytes	Annual
Cactaceae	*Opuntia ficus-indica* (L.) Mill	Phanerophytes	Perennial
Cistaceae	*Cistus albidus* L.	Chamephytes	Perennial
	*Cistus ladanifer* L.	Chamephytes	Perennial
Cupressaceae	*Tetraclinis articulata* (Vahl) Mast.	Phanerophytes	Perennial
	*Juniperus phoenicea* L.	Phanerophytes	Perennial
	*Juniperus oxycedrus* (L.) Cade	Phanerophytes	Perennial
Fabaceae	*Ulex europaeus* (L.) Ajonc, Lande	Phanerophytes	Perennial
Fagaceae	*Quercus ilex* L.	Phanerophyte	Perennial
Lamiaceae	*Marrubium vulgare* L.	Hemicryptophytes	Perennial
	*Lavandula angustifolia* Mill.	Chamephytes	Perennial
	*Lavandula dentata* L.	Chamephytes	Perennial
	*Thymus vulgaris* L.	Chamephytes	Perennial
	*Mentha pulegium* L.	Hemicryptophytes	Perennial
	*Mentha suaveolens* Ehrh.	Hemicryptophytes	Perennial
Mimosaceae	*Acacia retinodes* Schltdl.	Phanerophytes	Perennial
Moraceae	*Ficus carica* L.	Phanerophytes	Perennial
Myrtaceae	*Eucalyptus globulus* Labill.	Phanerophytes	Perennial
Oleaceae	*Olea europaea subsp. Europaea* L.	Phanerophytes	Perennial
	*Olea europea* L. *subsp. Europae* var. *sylvestris* (Mill) Lehr,	Phanerophytes	Perennial
Pinaceae	*Pinus pinaster* Aiton	Phanerophyte	Perennial
	*Pinus halepensis* Mill.	Phanerophytes	Perennial
	*Cedrus atlantica* (Manetti ex Endl.)	Phanerophytes	Perennial
Rhamnaceae	*Ziziphus jujuba* P. Miller	Phanerophytes	Perennial
	*Ziziphus lotus* (L.) Lam.	Phanerophytes	Perennial
Rosaceae	*Rosa damascena* Mill.	Phanerophytes	Perennial
Thymelaeaceae	*Daphne gnidium* L.	Phanerophytes	Perennial
Typhaceae	*Typha latifolia* L.	Geophytes	Perennial

**Table 5:** Summary statistics of LST and NDVI.

	LST	NDVI
Samples	36	36
Average	35.91	0.26
Std. deviation	7.65	0.42
Coef. of variation	21.32%	160.99%
Minimum	17.93	−0.62
Maximum	52.06	0.92
Extent	34.13	1.54
Std. skewness	−0.24	0.53
Std. Kurtosis	0.21	0.77

**Table 6:** Regression simple linear model.

Correlation Coefficient	−0.88
R^2^	78.21%
R^2^ (adjusted)	77.57%
Estimation of the standard deviation of the residue	3.62688
Mean absolute error	2.63072
Durbin-Watson statistic	0.406548 (*p* = 0.0000)
Analyze of variance *p*-value	0.0000

## Data Availability

The data used to support the findings of this study are available from the corresponding author upon request.

## Abbreviations

NDVI	Normalized Difference Vegetation Index
LST	Land surface temperature
